# Supplementary figures and images for: Comparison of the epidemiological and clinical fingerprints of Human Granulocytotropic Anaplasmosis and Human Monocytotropic Ehrlichiosis in the United States
Source: PLoS One. 2025 Nov 11;20(11):e0334957. doi: 10.1371/journal.pone.0334957 (PMC12604789; doi:10.1371/journal.pone.0334957)

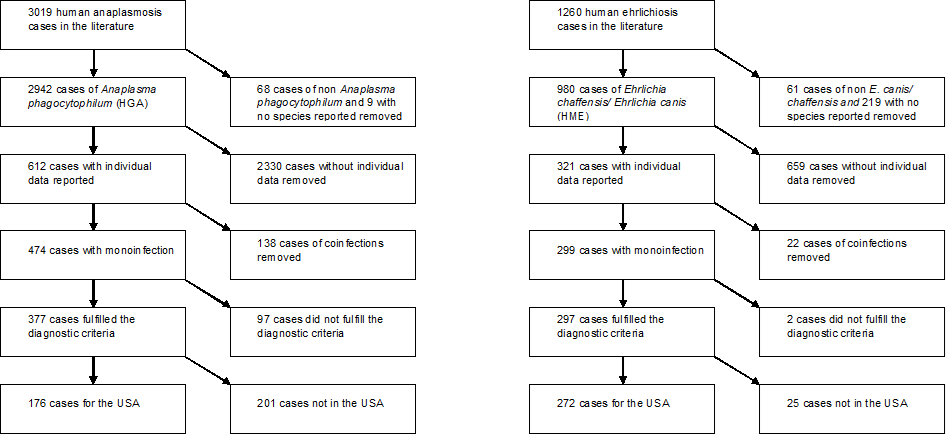

Supplement: S1 Fig — Allocation and selection of cases from the Schudel et al, and Gygax et al with the addition of selection for cases for the USA for analysis. (TIF) [file pone.0334957.s001.tif]

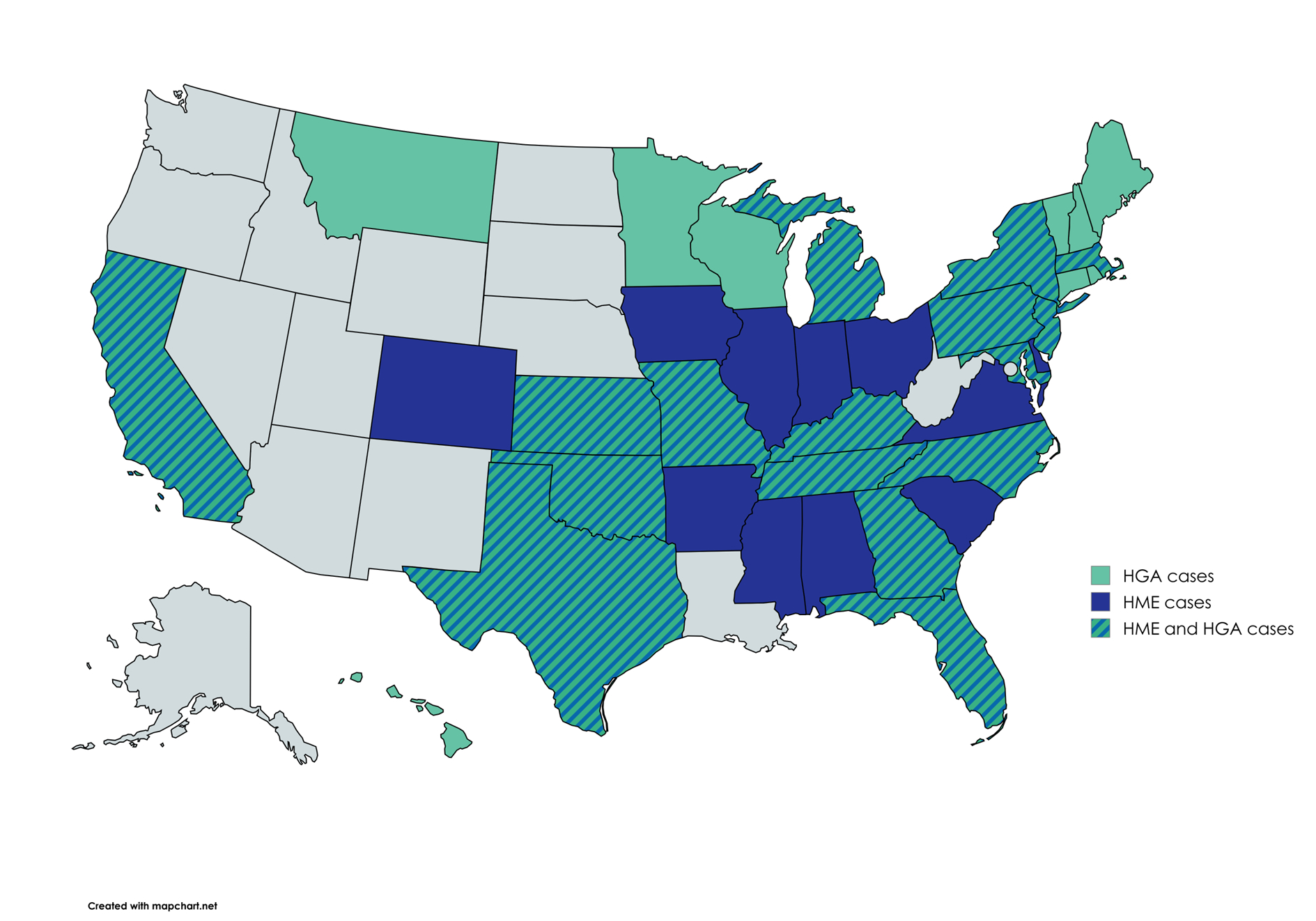

Supplement: S2 Fig — Geographic distribution of the analyzed 176 HGA and 272 HME cases. (TIF) [file pone.0334957.s002.tif]
