# Supplementary material for: Comparison of the epidemiological and clinical fingerprints of Human Granulocytotropic Anaplasmosis and Human Monocytotropic Ehrlichiosis in the United States
Source: PLoS One. 2025 Nov 11;20(11):e0334957. doi: 10.1371/journal.pone.0334957 (PMC12604789; doi:10.1371/journal.pone.0334957)
Supplement: Attachment — Submitted filename: reviewer comments_23082025.docx [file pone.0334957.s005.docx]

We cordially want to thank the two reviewers for taking their time to review our manuscript, their critical eyes, and their constructive remarks and input to the manuscript.

In the following, we will address the points listed by the two reviewers in chronological order and also address what we adapted in the manuscript following the comments from both reviewers.

**Reviewer #1:**

Thank you to the authors for their revision of the manuscript and response to reviewer comments. The clarity of the manuscript has improved. I only have a few minor comments.
Line 76/Fig 1. There appears to be a discrepancy between the text (both diseases are notifiable in all 50 US states) and Figure 1 (several states are indicated as not notifiable). Please reconcile.

**RESPONSE:** Thank you for pointing this out. We changed the “not notifiable” to “no data”, which is much more appropriate.

Figure 1. Given how similar the ranges in incidence are between the panels, I would suggest using the same cut points for color. The readers can compare relative magnitudes between panels.

**RESPONSE:** We do understand the request, however the reason for these different cutoffs, is that the map is a 1:1 recreation of the originals used by CDC including the cutoffs. The recreation was done because the original did not have the required dpi quality, and we would prefer to keep them as an identical copy of the original.

Line 144. The ‘p’ is missing (i.e., should read p<0.001).

**RESPONSE:** Corrected.

Line 150. There is a typo for “such”.

**RESPONSE:** Corrected.

Table 2. I suggest the inclusion of a footnote explaining the reason for differences in denominators within columns (i.e., lack of standardized reporting). The authors explained this in their response to reviewers, but did not include this information in the revised document. I would also suggest the authors add this caveat explicitly to the limitations. Differential missingness of reported symptoms across diseases could bias the estimates proportions.

**RESPONSE:** This is a good point, thank you very much. We added the line in the footnote. Additionally, we added the line “the differential missingness of reported symptoms across the two diseases could bias the estimated proportions” at line 254 and “or contradict” at line 257, to put more emphasis on the unfortunate lack of standardized reporting.

**Reviewer #2:**

I would like to express my appreciation to the authors for their revisions. While not all of my concerns have been fully addressed, I wish to respect the authors’ autonomy, and I find the minor comments they chose not to incorporate to be acceptable.

However, one major concern remains regarding the discussion of Anaplasma encephalitis. The authors are quick to critique the case report and literature review conducted by researchers at the Mayo Clinic, yet they do not provide any substantiating evidence for the 29 cases cited in their results. Furthermore, the authors acknowledge the possibility of co-infection in these cases, which raises additional questions regarding the validity of the findings.

In my view, there are two appropriate courses of action: either (1) eliminate the discussion and corresponding results related to encephalitis entirely, or (2) retain the section but support it with a thorough and detailed review of the literature, specifically addressing all 29 cases included in the results.

**RESPONSE:** We would like to point out, that we are not quick to criticize the literature review and not at all the case report itself, but the fact is that the reference used for .” Neurologic manifestations of anaplasmosis are less common than in other tick-borne diseases such as ehrlichiosis, Lyme disease, and Powassan virus infection [[2](https://www.mdpi.com/2036-7449/15/4/35#B2-idr-15-00035),[6](https://www.mdpi.com/2036-7449/15/4/35#B6-idr-15-00035)]” and “Neurologic symptoms and complications in HGA are rare [2]” where specifically ref 2 is “Ismail, N.; McBride, J.W. Tick-Borne Emerging Infections.” and the paragraph on“Human Granulocytic Anaplasmosis Neurologic Features” in Ismael et al on neurological symptoms cites:

- Choi KS, Scorpio DG, Barat NC. Msp2 variation in Anaplasma phagocytophilum in vivo does not stimulate T cell immune responses or interferon-gamma production. FEMS Immunol Med Microbiol 2007;49:374–86.
- Horowitz HW, Marks SJ, Weintraub M, et al. Brachial plexopathy associated with human granulocytic ehrlichiosis. Neurology 1996;46:1026–9.
- Akkoyunlu M, Fikrig E. Gamma interferon dominates the murine cytokine response to the agent of human granulocytic ehrlichiosis and helps to control the degree of early rickettsemia. Infect Immun 2000;68:1827–33.

This might have been citation oversight by Ismael et al. Nevertheless, none of these three citations provide any prevalence or incidence data on the involvement of the CNS. Therefore, this reference does not hold up.

We do acknowledge that ref 6 “Bakken, J.S.; Dumler, J.S. Human Granulocytic Anaplasmosis” references the respective data, with 5 cases over 6 years from Dahlgren et al. However, they had access to the national database of the CDC, whereas published case reports can suffer from a “this is interesting, let’s publish it” bias as seen by the much lower numbers of published cases vs. the national database. Additionally, the amount of our encephalitis cases is over 30-year period which roughly corresponds to the amount found by Dahlgren et al. over their 6 years, i.e. a case per year. Furthermore, we do not claim that encephalitis is common in HGA, but that it may be more frequent in HGA when compared to HME, although still an overall small number.

We reviewed the cases, excluded those with other potential causes which reduced the immunocompetent cases to 22, which would be a p of 0.04, primarily based on confusion, as most additional data was lacking (liquor, EEGs, etc.) for a more solid definition of encephalitis (based on the 2013 criteria).

Because of the long-time frame and potentially different definitions of encephalitis and the often missing additional information in the case reports, we decided to remove the encephalitis line from the results, the table and the discussion.

We added this line to the discussion “Unfortunately, the data from these case reports were insufficient to provide a reliable estimate on the difference of cases with possible encephalitis between the two diseases, and therefore this point remains unresolved.” for clarification.
